# *Helicobacter pylori* promotes angiogenesis depending on Wnt/beta-catenin-mediated vascular endothelial growth factor via the cyclooxygenase-2 pathway in gastric cancer

**DOI:** 10.1186/s12885-016-2351-9

**Published:** 2016-05-19

**Authors:** Ningning Liu, Ning Zhou, Ni Chai, Xuan Liu, Haili Jiang, Qiong Wu, Qi Li

**Affiliations:** Department of Medical Oncology, Shuguang Hospital, Shanghai University of Traditional Chinese Medicine, No. 528 Zhangheng Road, Shanghai, 201203 P. R. China

**Keywords:** *Helicobacter pylori*, Gastric cancer, Vascular endothelial growth factor, Cyclooxygenase 2, Wnt/beta-catenin, Angiogenesis

## Abstract

**Background:**

*Helicobacter pylori* is an important pathogenic factor in gastric carcinogenesis. Angiogenesis (i.e., the growth of new blood vessels) is closely associated with the incidence and development of gastric cancer. Our previous study found that COX-2 stimulates gastric cancer cells to induce expression of the angiogenic growth factor VEGF through an unknown mechanism. Therefore, the aim of this study was to clarify the role of angiogenesis in *H. pylori*-induced gastric cancer development.

**Methods:**

To clarify the relationship between *H. pylori* infection and angiogenesis, we first investigated *H. pylori* colonization, COX-2, VEGF, beta-catenin expression, and microvessel density (MVD) in gastric cancer tissues from 106 patients. In addition, COX-2, phospho-beta-catenin, and beta-catenin expression were measured by western blotting, and VEGF expression was measured by ELISA in *H. pylori*-infected SGC7901 and MKN45 human gastric cancer cells.

**Results:**

*H. pylori* colonization occurred in 36.8 % of gastric carcinoma samples. Furthermore, COX-2, beta-catenin, and VEGF expression, and MVD were significantly higher in *H. pylori*-positive gastric cancer tissues than in *H. pylori*-negative gastric cancer tissues (*P* < 0.01). *H. pylori* infection was not related to sex or age in gastric cancer patients, but correlated with the depth of tumor invasion, lymph node metastasis, and tumor–node–metastasis stage (*P* < 0.05) and correlated with the COX-2 expression and beta-catenin expression(*P* < 0.01). Further cell experiments confirmed that *H. pylori* infection upregulated VEGF in vitro. Further analysis revealed that *H. pylori*-induced VEGF expression was mediated by COX-2 via activation of the Wnt/beta-catenin pathway.

**Conclusions:**

The COX-2/Wnt/beta-catenin/VEGF pathway plays an important role in *H. pylori*-associated gastric cancer development. The COX-2/Wnt/beta-catenin pathway is therefore a novel therapeutic target for *H. pylori*-associated gastric cancers.

## Background

*Helicobacter pylori* is a Gram-negative, spiral bacillus that infects approximately half the world’s population and induces chronic inflammation of the gastric mucosa, contributing to the development of peptic ulcer and gastric malignancies [1, 2]. *H. pylori* has been classified as a class I carcinogen by the International Agency for Research on Cancer (IARC) and World Health Organization (WHO) [3]. However, the pathogenesis of *H. pylori* infection–induced gastric cancer has not been fully elucidated.

Angiogenesis is already present in early gastric cancer, and its development requires a unique tumor phenotype and necessary ingredients. As the cancer progresses toward more advanced stages, angiogenesis becomes more pronounced. Angiogenesis and the occurrence and development of gastric cancer are closely related [4]. Angiogenesis is a key step in tumor growth and metastasis [5]. Neovascularization not only provides nutrients and oxygen to the tumor cells, and carries away metabolic waste, but it also stimulates tumor growth through autocrine or paracrine modes of action. It is a complex process of angiogenesis, which is co-regulated by angiogenic and anti-angiogenic factors. Gastric cancer cells can produce a variety of proangiogenic growth factors [6], and vascular endothelial growth factor (VEGF) is the strongest and the most specific angiogenic growth factor. VEGF plays a major role in the multistep process of angiogenesis stimulation and is closely related to the development of gastric cancer [7]. Moreover, VEGF plays a pivotal role in tumor-associated microvascular angiogenesis [8] and has been demonstrated to be overexpressed in human gastric carcinomas [9–11]. Although there have been numerous reports on *H. pylori* infection influencing angiogenesis in gastric cancer, the exact mechanism remains unclear.

COX is a key rate-limiting enzyme in the conversion of arachidonic acid to prostanoids and thromboxanes; it exists in two forms, cyclooxygenase 1 (COX-1) and COX-2 [12, 13]. COX-1 is responsible for maintaining normal physiological function; it is expressed constitutively in most tissues. In contrast, COX-2 is an early response gene induced by growth factors, proinflammatory cytokines, tumor promoters, and bacterial toxins [14–16]. We earlier demonstrated that *H. pylori* can upregulate COX-2 via the p38 mitogen-activated protein kinase (MAPK)/activating transcription factor-2 (ATF-2) signaling pathway in MKN45 gastric cancer cells [17]. Caputo *et al*. [18] reported that *H. pylori* induced VEGF upregulation in MKN28 gastric cancer cells, which might be mediated by COX-2. Moreover, research shows that that *H. pylori* infection influences angiogenesis in gastric cancer patients [19]. Considering these results, it is reasonable to believe that COX-2 might play a role in VEGF upregulation in *H. pylori*-infected gastric cancers.

The Wnt/beta-catenin pathway is commonly activated during carcinogenesis [20]. In the classical Wnt signaling pathway, Wnt binding to its Fz receptor inactivates the beta-catenin destructive complex comprising adenomatous polyposis coli (APC), axin, and glycogen synthase kinase-3 beta (GSK3beta). Beta-catenin then disassociates from the complex, translocates into the nucleus, and binds to members of the lymphoid-enhancing factor/T-cell factors (Tcf/Lef) family that activate target gene transcription when the Wnt pathway is activated [21]. Normally, beta-catenin phosphorylation maintains the complex in a stable state, and unphosphorylated beta-catenin enters into the nucleus when Wnt pathway is activated. The Wnt/beta-catenin pathway is important for angiogenesis, and beta-catenin is associated with COX-2 overexpression [22] and angiogenesis [23]. However, whether the Wnt/beta-catenin pathway plays a role in *H. pylori*-induced angiogenesis is unclear.

In the present study, we aimed to investigate whether the Wnt/beta-catenin pathway is involved in *H. pylori*-induced upregulation of angiogenesis in gastric cancer.

## Methods

### *H. pylori* culture

The *H. pylori* cagA- and vacA-positive standard strain NCTC11637 was obtained from the Institute of Digestive Diseases, Renji Hospital, Shanghai Jiao Tong University, Shanghai, China. *H. pylori* was cultured on Columbia agar (Oxoid, Basingstoke Hampshire, UK) plates containing 5 % sheep blood and incubated at 37 °C under microaerophilic conditions for 48–72 h. Colonies were identified as *H. pylori* by Gram staining, morphology, and positive oxidase, catalase, and urease activities. Bacteria were suspended in phosphate-buffered saline (PBS) and the density was estimated by spectrophotometry (OD_600 nm_) and microscopic observation.

### Immunohistochemical staining of COX-2, beta-catenin, VEGF, and CD34 in human gastric carcinoma tissues

A total of 106 different formalin-fixed, paraffin-embedded gastric cancer tissue samples and adjacent normal tissues were obtained from Shuguang Hospital, Shanghai University of Traditional Chinese Medicine. The use of all human tissue samples was approved by the Institutional Review Board of Shuguang Hospital, which is affiliated with Shanghai University of Traditional Chinese Medicine. Informed consent was obtained from every patient for the use of all human tissues used in this study. First, tissue samples were stained with Giemsa to determine the presence of *H. pylori* infection. Next, using standard methods, COX-2, beta-catenin, VEGF, and CD34 were detected immunohistochemically. Briefly, tissues were embedded in paraffin and 4-μm sections were cut, deparaffinized in xylene, and dehydrated through a graded alcohol series. Tissue sections were subjected to peroxidase clearance, antigen retrieval, and blocking of non-specific binding sites. Sections were first incubated with primary antibody (rabbit polyclonal antibodies against CD34, COX-2, beta-catenin, and VEGF (Abcam, Cambridge, MA, USA), followed by EnVision secondary antibody (Dako, Glostrup, Denmark). Sections were counterstained with hematoxylin. PBS served as a negative control for primary antibody. Staining intensity was assessed in each specimen on a scale of 0–3: 0, no staining; 1, weak staining; 2, moderate staining; and 3, strong staining.

### Immunohistochemical analysis of the MVD

According to Weidner [24], areas of highest neovascularization were found by scanning tumor sections at low-power (×40) magnification. After the area of highest neovascularization was identified, individual microvessel counts were made in a single high-power (×200) magnification field. Three different visual fields were selected for microvessel counting, and the mean value was recorded. Brown-staining endothelial cells or endothelial cell clusters were considered as single, countable microvessels.

### Cell culture and reagents

SGC7901 and MKN45 gastric cancer cells were obtained from the Institute of Digestive Diseases, Renji Hospital of Shanghai Jiao Tong University, Shanghai, China, and cultured in RPMI 1640(Gibco, Thermo Fisher Scientific Inc, Waltham, MA, USA) containing 10 % (v/v) fetal bovine serum (Gibco, Thermo Fisher Scientific Inc, Waltham, MA, USA) and 1 % penicillin and streptomycin (North China Pharmaceutical Company, Shijiazhuang, China). Cells were plated in 6-well plates and grown to confluency. FH535, a beta-catenin-specific inhibitor, was obtained from Cell Signaling (Beverly, MA, USA). All cells were grown in a humidified incubator containing 5 % CO_2_ at 37 °C.

### Real-time fluorogenic quantitative polymerase chain reaction

#### RNA isolation

Total cellular RNA was prepared using RNAisol reagent (TaKaRa Biotechnology, Dalian, China) according to the manufacturer’s instructions. RNAisol (1 ml) was added to each sample and incubated for 5 min at room temperature. Next, 200 μl chloroform was added and samples were shaken for 15 s and incubated at room temperature for 2-3 min and then centrifuged at 12,000 *g* for 15 min at 4 °C after formation of a biphasic solution. For RNA precipitation, the aqueous phase (top) was transferred to a new tube and 500 μl isopropanol was added. Samples were incubated at room temperature for 5-10 min and then centrifuged at 12,000 *g* for 15 min at 4 °C, after which a pellet was visible. After supernatant removal, 1000 μl of 75 % ethanol was added to wash the RNA pellet; this was vortexed and centrifuged at 8000 *g* for 5 min at 4 °C. After the ethanol was carefully removed by pipetting, the RNA pellet was air-dried for 5-10 min and then dissolved in diethylpyrocarbonate-treated water with vortexing. RNA quality was verified by agarose gel electrophoresis and visualization of 28S and 18S ribosomal RNA. RNA was quantified by spectrophotometry (OD_260/280 nm_). RNA was then immediately frozen at −70 °C.

#### cDNA synthesis and real-time quantitative analysis

Reverse transcription was conducted using a PrimeScript RT-PCR Kit (TaKaRa Biotechnology, Dalian, China). Total RNA (1 μg) was used as a template for cDNA synthesis. Briefly, reverse transcription was carried out in a 20-μl solution including 4 μl 5× buffer, 1 μl oligo dT primer, 1 μl random 6-mers, 1 μl PrimeScript RT Enzyme Mix, and RNAse-free deionized H_2_O. Reverse transcription incubation conditions were 37 °C for 15 min and 85 °C for 5 s The resultant cDNA was stored at −20 °C until it was used for real-time quantitative polymerase chain reaction (PCR). Real-time PCR reactions were carried out using the ABI7300 Fast Real-Time PCR System (PE Biosystems, Foster City, CA, USA) using a PrimeScript RT-PCR Kit according to the manufacturer’s instructions. Primers and probes for human *GAPDH*, *VEGF*, and *COX2* were designed and synthesized by Shanghai Shanjing Biotechnology (Shanghai, China) with FAM (6-carboxy-fluo-rescein-phosphoramidite)-labeled 5′ ends and TAMPA (carboxy-tetramethyl-rhodamine)-labeled 3′ ends. Primer and probe sequences were: human *GAPDH*-forward, 5′-CCACTCCTCCACCTTTGAC-3′; human *GAPDH*-reverse, 5′-ACCCTGTTGCTGTAGCCA-3′; *GAPDH* probe, 5′-TTGCCCTCAACGACCACTTTGTC-3′; human *VEGF*-forward, 5′-GGCCTCCGAAACCATGAACT-3′, human *VEGF*-reverse, 5′-ACCCTGTTGCTGTAGCCA-3′; and *VEGF* probe, 5′-TGTCTT GGGTGCATTGGAGC-3′. Briefly, each PCR was performed in a 20-μl reaction volume comprising 10 μl Premix EX Taq, 0.4 μl Rox reference dye, 0.4 μl each primer, 0.8 μl TaqMan probe, 6 μl deionized H_2_O and 2 μl cDNA. PCR cycling conditions were 95 °C for 10 s, followed by 40 cycles of 95 °C for 5 s (denaturation) and 60 °C for 31 s (annealing/extension). Each reaction was performed in triplicate, and data were analyzed by the 2^−∆∆Ct^ method for comparing relative expression levels. *GAPDH* mRNA was used to normalize RNA levels from the various samples and mRNA expression was expressed as relative to the basal level without *H. pylori* stimulation.

### Western blot analysis

Following treatment, cells were washed twice with ice-cold PBS and then protease inhibitors (Roch, Basel, Switzerland) were added. Cells were then scraped off the dish, and then cytoplasmic and nuclear fractions were prepared using a protein extraction kit (Fermentas, Waltham, MA, USA). Cell lysis buffer, nuclei washing buffer, and other reagent buffers were added to separate cytosolic proteins and nuclear proteins. The protein concentration in extracts was determined by bicinchoninic acid protein assay using a commercial kit (BCA Protein Assay Reagent; Merck, Whitehouse Station, NJ, USA). Protein samples were separated by 10 % SDS-PAGE and transferred to PVDF membrane. The membrane was incubated in blocking buffer (10 mmol/l Tris, pH 7.5, 100 mmol/l NaCl, 0.1 % Tween 20), containing 5 % nonfat powdered milk for 1 h. The membrane was then incubated with anti-phospho-beta-catenin or anti- beta-catenin polyclonal antibody (1:500; Cell Signaling Technology, USA). Following overnight incubation at 4 °C, blots were washed three times in TBS-Tween (0.05 %) solution and incubated with goat anti-rabbit antibodies conjugated to horseradish peroxidase (HRP) for 1 h at room temperature before visualizing using the Pierce ECL kit (Thermo Fisher Scientific Inc, Waltham, MA, USA). Results were analyzed by Image J software (NIH Image).

### Enzyme-linked immunosorbent assay

Cell culture supernatant samples were collected and clarified at 3000 *g* for 5 min. ELISA was performed according to the manufacturer’s protocol. Briefly, microtiter plates were incubated with 100 μl samples at 37 °C for 120 min. After five washes in 10 mM PBS, plates were incubated with 100 μl anti-VEGF primary antibody labeled with biotin (from the ELISA kit) at 37 °C for 60 min. After five rinses with 10 mM PBS, 100 μl avidin-biotin-peroxidase complex was added to wells and incubated at 37 °C for 30 min. After extensive rinsing, 100 μl/well TMB Microwell Substrate and was added and plates were incubated in the dark at 37 °C for 15 min. The reaction was then stopped with 100 μl TMB stop solution and OD_450 nm_ values were obtained within 30 min using a microplate reader. Finally, protein concentrations were determined from OD values using a calibration curve.

### Statistical analysis

Statistical analyses were performed using the Statistical Package for the Social Sciences (SPSS version 19.0). Statistical significance was determined by *t* tests and one-way ANOVA followed by Fisher’s least significant difference test and differences in rates were determined by the chi-squared test. Data are presented as means ± SE and a *P* value of <0.05 was considered statistically significant.

## Results

### *H. Pylori* infection correlates with COX-2, VEGF, and beta-catenin upregulation and angiogenesis in gastric cancer

**Fig. 1:**
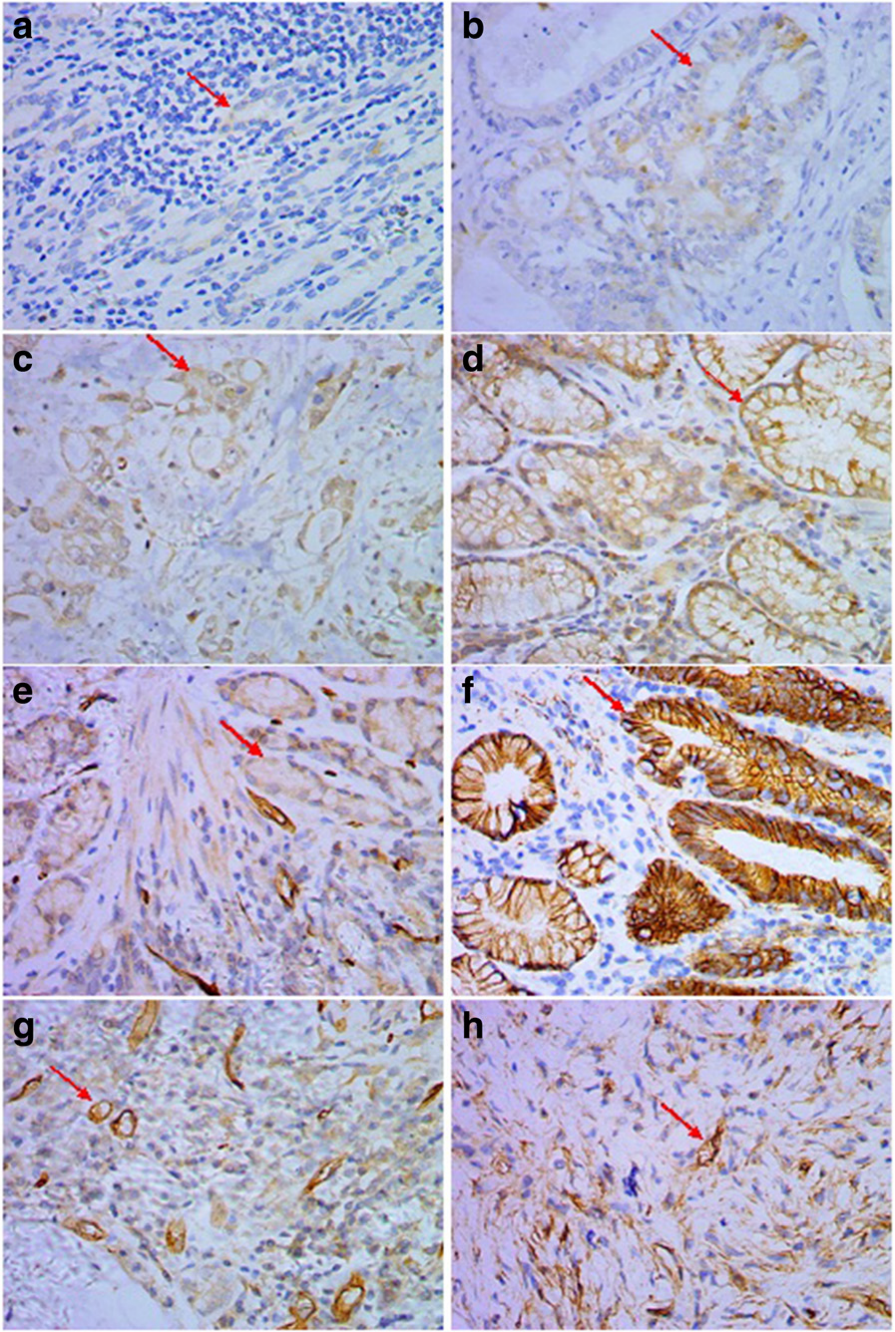
Immunohistochemical assessment of COX-2, beta-catenin, and VEGF expression, and MVD in human gastric cancers. **a** COX-2 expression in *H. pylori*-negative gastric cancer tissues. **b**. COX-2 expression in *H. pylori*-positive gastric cancer tissues. **c** VEGF expression in *H. pylori*-negative gastric cancer tissues. **d** VEGF expression in *H. pylori*-positive gastric cancer tissues. **e** Beta-catenin expression in *H. pylori*-negative gastric cancer tissues. **f**. Beta-catenin expression in *H. pylori*-positive gastric cancer tissues. **g** MVD in *H. pylori*-negative gastric cancer tissues. **h** MVD in *H. pylori*-positive gastric cancer tissues. Red arrows indicate positive staining. Magnification, ×200. NOTE: Specimens were obtained from 106 patients who underwent major surgical resection for gastric cancer with no preoperative chemotherapy and radiotherapy between February 2009 and December 2014 at the Department of Surgery, Shuguang Hospital (affiliated with Shanghai University of Traditional Chinese Medicine, Shanghai, PR China)

To investigate a correlation between *H. pylori* and gastric cancer, we first observed *H. pylori* colonization in 106 gastric cancer tissues. The results showed that *H. pylori* colonization is present in 36.8 % (39/106) of gastric carcinoma samples. Immunohistochemical analysis of gastric carcinomas and matched normal mucosa showed that the mean staining intensity of COX-2 in *H. pylori*-positive gastric carcinoma was 2.26 ± 0.17, significantly higher than in *H. pylori*-negative gastric cancer tissues (0.63 ± 0.16, *P* < 0.01; Fig. 1a, b). Similarly, the mean VEGF staining intensity in *H. pylori*-positive gastric carcinoma was significantly higher than in *H. pylori*-negative tissues (2.65 ± 0.11 vs 0.85 ± 0.06, *P* < 0.01; Fig. 1c, d). *H. pylori*-positive gastric cancers had a significantly higher staining intensity for beta-catenin in the gastric mucosa compared with *H. pylori*-negative gastric cancers (1.95 ± 0.09 vs 0.45 ± 0.075, *P* < 0.01; Fig. 1e, f). Blood vessel counts in *H. pylori*-positive and *H. pylori*-negative gastric cancer tissues showed that the microvessel density (MVD) was 42.9 ± 4.9 and 18.1 ± 3.5, respectively (*P* < 0.01; Fig. 1g, h). The presence of *H. pylori* infection in gastric cancer was not related to sex or age, but correlated with the depth of tumor invasion, lymph node metastasis, and tumor–node–metastasis stage(*P* < 0.05 Table 1) and correlated with the COX-2 expression and beta-catenin expression(*P* < 0.01 Table 1). These results suggest that *H. pylori* infection promotes COX-2, VEGF, and beta-catenin upregulation and increases MVD in gastric cancer, which might play an important role in gastric cancer development.

**Table 1:** Relationship between *H. pylori* infection, COX-2 and beta-catenin expression, and clinicopathological features in gastric cancer

Parameters	*N*	*H. pylori*-positive	*H. pylori*-negative	*P* value
Age/year				
< 65	66	25	41	>0.05
> 65	40	14	26	
Sex				
Male	68	27	41	>0.05
Female	38	12	26	
T classification				
T_1–2_	36	8	28	<0.05
T_3–4_	70	31	39	
Lymph node metastasis				
Yes	76	33	43	<0.05
No	30	6	24	
TNM stage				
I II	37	8	29	<0.05
III IV	69	31	38	
COX-2 expression				
Low	35	5	30	<0.01
High	71	34	37	
Beta-catenin expression				
Low	41	7	34	<0.01
High	65	32	33	

### Effect of *H. pylori* on VEGF mRNA and protein levels in SGC7901 and MKN45 cells

**Fig. 2:**
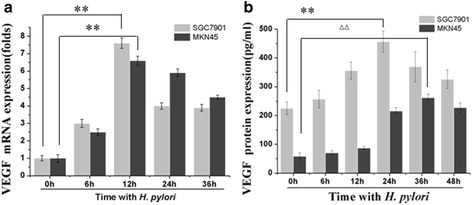
Time-dependent VEGF induction by *H. pylori* in SGC7901 and MKN45 cells. **a** *VEGF* mRNA expression increased in *H. pylori*-treated cells. Confluent SGC7901 and MKN45 cells were incubated with 100 *H. pylori* bacteria/cell for 0, 6, 12, 24, and 36 h and then analyzed by real-time quantitative PCR to determine *VEGF* mRNA expression relative to *GAPDH* mRNA. **P < 0.01, 12 h versus 0 h in SGC7901 and MKN45 cells. **b** VEGF protein content was increased in *H. pylori*-treated cells. SGC7901 and MKN45 cells were incubated with *H. pylori* for 0, 6, 12, 24, 36, and 48 h, and then analyzed by ELISA to determine VEGF protein levels. **P < 0.01, 24 h versus 0 h in SGC7901 cells; ^△△^ *P* < 0.01, 36 h versus 0 h in MKN45 cells

To further confirm the VEGF upregulation in *H. pylori*-infected gastric carcinomas, we investigated the effect of *H. pylori* infection in gastric cancer cells on VEGF expression in vitro. After 6, 12, and 24 h incubation with *H. pylori* NCTC11637 strain, there was differential upregulation of *VEGF* mRNA in SGC7901 and MKN45 cells (Fig. 2a). To assess whether *H. pylori* upregulation of VEGF also occurred at the protein level, we measured VEGF at 6, 12, 24, 36, and 48 h in *H. pylori*-treated or untreated (control) MKN45 cells by ELISA (Fig. 2b). These data further suggest that the VEGF is upregulated by *H. pylori* infecting SGC7901 and MKN45 cells.

### *H. pylori* upregulates VEGF via COX-2

**Fig. 3:**
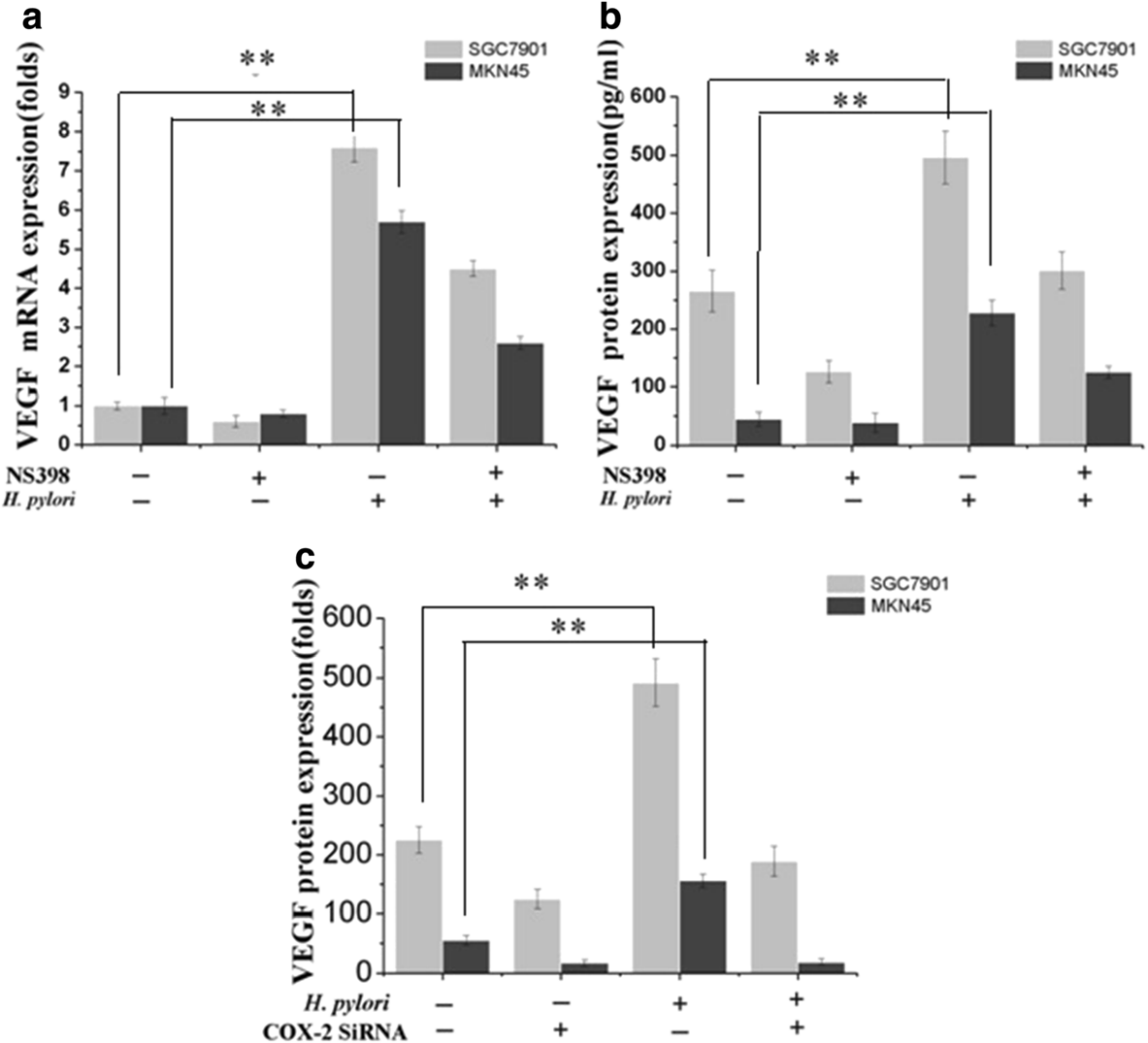
COX-2 inhibition attenuates *H. pylori-*induced VEGF upregulation in SGC7901 and MKN45 cells. **a** The COX-2 inhibitor NS398 attenuated *H. pylori*-dependent *VEGF* mRNA induction. Confluent SGC7901 and MKN45 cells were pretreated with 50 μM NS398 for 2 h prior to culture with or without *H. pylori* for 12 h. *VEGF* mRNA expression relative to *GAPDH* mRNA was determined by quantitative RT-PCR. **P < 0.01 for *H. pylori*-infected vs control cells. **b** NS398 attenuated *H. pylori* induction of VEGF protein. SGC7901 and MKN45 cells were pretreated with 50 μM NS-398 for 2 h prior to culture with or without *H. pylori* for 24 h and 36 h. The culture supernatant was then analyzed by ELISA to determine VEGF protein content. **P < 0.01 for *H. pylori*-infected vs control SGC7901 and MKN45 cells. **c** RNAi-mediated *COX2* inhibition blocked VEGF upregulation by *H. pylori*. Confluent SGC7901 and MKN45 cells were infected with pFU-GW-COX-2-shRNA for 72 h with or without *H. pylori* treatment for 48 h. VEGF protein content in culture supernatant was then measured by ELISA. **P < 0.01 for *H. pylori*-infected vs control cells

We previously reported that *H. pylori* upregulates COX-2 expression in vitro. To further verify whether VEGF expression is influenced by incubation with the selective COX-2 inhibitor NS398, real-time fluorogenic quantitative (RFQ) PCR analysis and western blotting were performed to measure VEGF mRNA and protein levels in SGC7901 and MKN45 cells. As shown in Fig. 3a and b, NS398 treatment of did not obviously affect basal VEGF mRNA or protein levels compared with the control group. Interestingly, higher VEGF levels were observed in SGC7901 and MKN45 cells infected with *H. pylori*; however, this was downregulated by COX-2 inhibition.

In our previous studies, the lenti-virus based RNAi of COX-2(pFU-GW-COX-2-shRNA) was constructed to suppress the endogenous COX-2 specially [25]. To further examine the effect of COX-2 on VEGF expression, we infected human gastric cancer SGC7901 and MKN45 cells with the lpFU-GW-COX-2-shRNA to suppress COX-2 expression. RFQ-PCR and western blotting showed that COX-2 gene silencing significantly decreased COX-2 mRNA and protein levels in SGC7901 and MKN45 cells. COX-2 gene silencing or COX-2 pathway inhibition also reduced VEGF levels (Fig. 3c). Considered together, our findings suggest that *H. pylori* upregulates VEGF in SGC7901 and MKN45 cells via increasing COX-2 expression.

### Activation of the Wnt/beta-catenin pathway by *H. pylori* infection

**Fig. 4:**
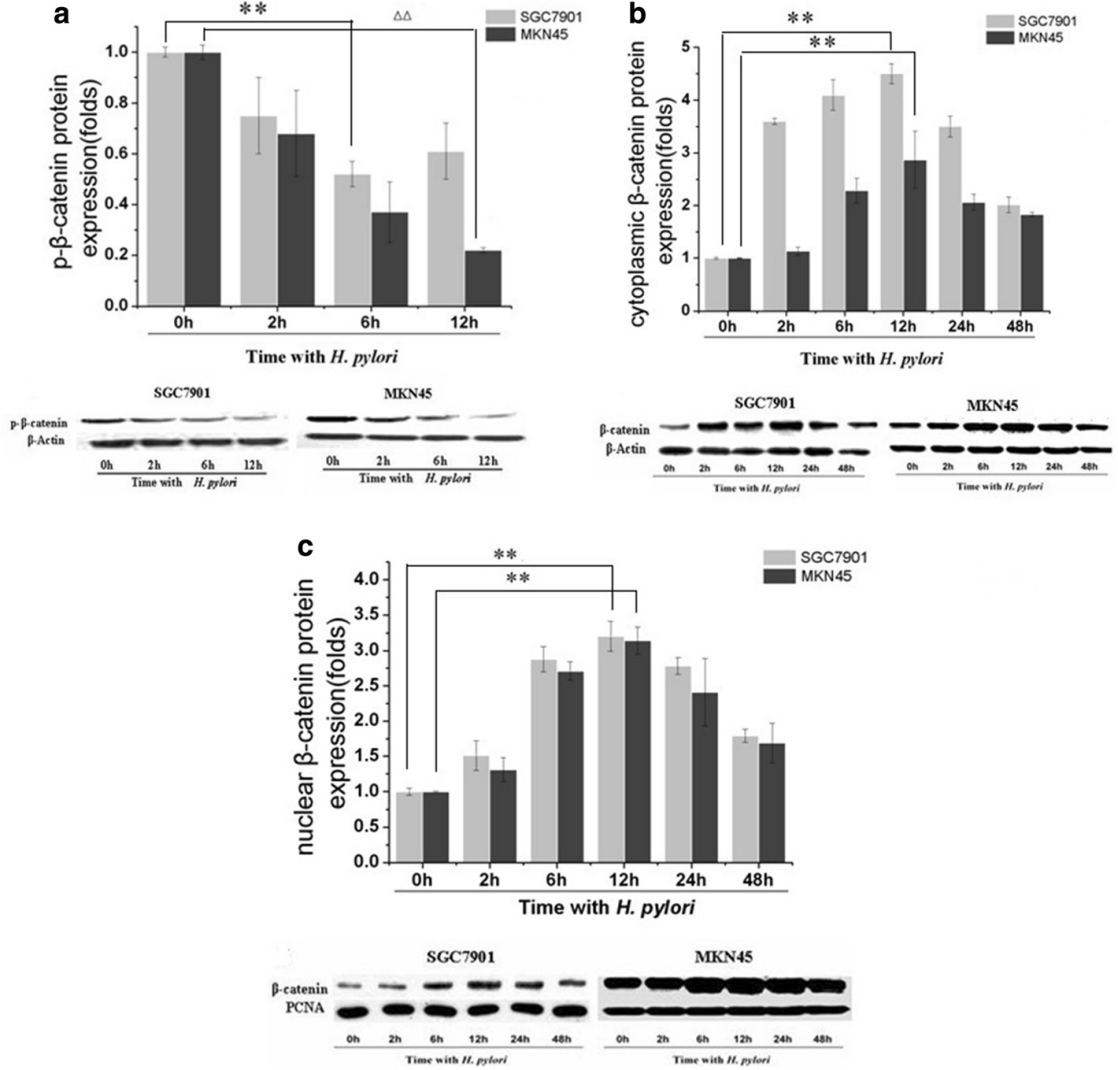
*H. pylori* affects phospho-beta-catenin and unphosphorylated beta-catenin protein levels in SGC7901 and MKN45 cells. Confluent SGC7901 and MKN45 gastric cancer cells were incubated with *H. pylori* at 100 bacteria/cell for 2, 6, 12, 24, and 48 h, and then p-beta-catenin and unphosphorylated beta-catenin protein levels were determined in total cellular protein extracts by western blotting. Blots were stripped and reprobed with beta-actin or PCNA to show equal protein loading. The experiment was performed for three times with similar results. **a** *H. pylori* attenuates cytoplasmic p-beta-catenin protein levels in SGC7901 and MKN45 cells. **P < 0.01 for 6 h versus 0 h in SGC7901 cells; ^△△^ *P* < 0.01 for 12 h versus 0 h in MKN45 cells. **b** *H. pylori* induces cytoplasmic beta-catenin accumulation in MKN45 cells. **P < 0.01 for 12 h versus 0 h in SGC7901 and MKN45 cells. **c** *H. pylori* induces nuclear beta-catenin accumulation in SGC7901 and MKN45 cells. **P < 0.01 for 12 h versus 0 h in SGC7901 and MKN45 cells

To identify whether the Wnt/beta-catenin pathway is involved in VEGF upregulation after *H. pylori* infection, we initially observed the effects of *H. pylori* infection on beta-catenin levels in SGC7901 and MKN45 cells. Western blot analysis with phospho-specific antibodies showed a time-dependent decrease in beta-catenin phosphorylation in the cytoplasm. However, *H. pylori* induced cytoplasmic and nuclear beta-catenin accumulation, showing that *H. pylori* infection could cause nuclear translocation of beta-catenin (Fig. 4). This phenomenon demonstrated that Wnt/beta-catenin might contribute to the *H. pylori*-induced VEGF transcription.

### Blocking Wnt/beta-catenin attenuates *H. pylori-*induced VEGF upregulation in SGC7901 and MKN45 cells

**Fig. 5:**
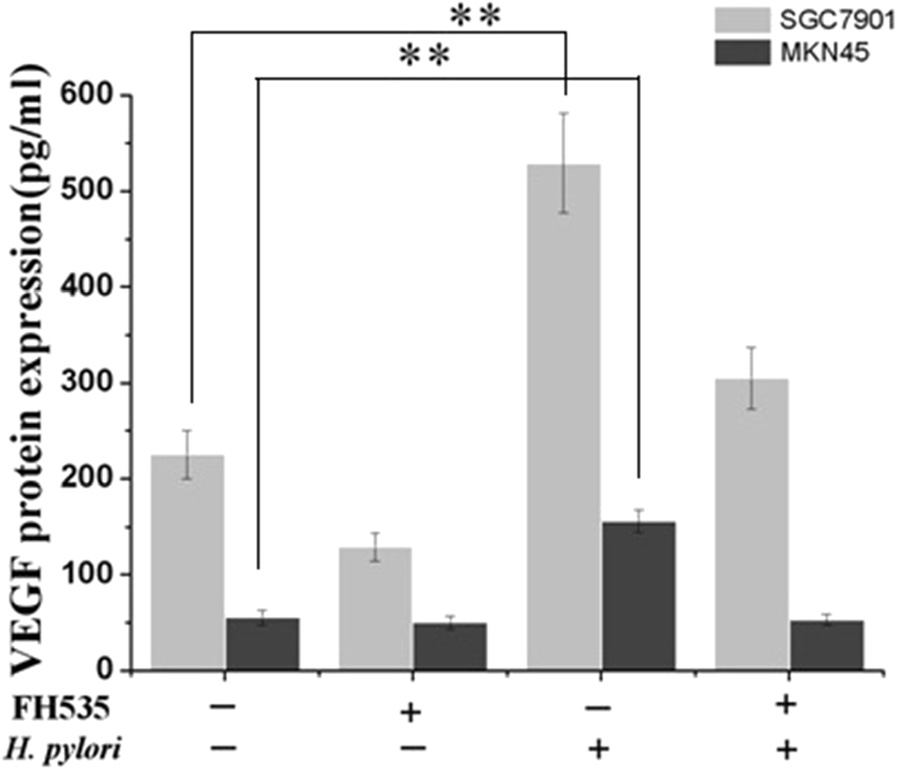
Beta-catenin inhibition attenuates *H. pylori*-induced VEGF upregulation in SGC7901 and MKN45 cells. Confluent SGC7901 and MKN45 cells were pretreated with 20 μM FH535 for 2 h prior to culture with or without *H. pylori* for 12 h. Relative cellular VEGF protein levels were then measured by ELISA. **P < 0.01 for *H. pylori*-infected versus control cells

Because *H. pylori* clearly induced the translocation of beta-catenin, additional studies were carried out with inhibitors of beta-catenin to determine the significance of Wnt/beta-catenin signaling pathways in *H. pylori*-induced VEGF upregulation. We found that *H. pylori*-induced VEGF mRNA and protein upregulation was partially blocked by FH535 (a specific inhibitor of beta-catenin activity, 20 μM; Fig. 5). Thus, the Wnt/beta-catenin signaling pathway might be primarily responsible for *H. pylori*-induced VEGF upregulation in SGC7901 and MKN45 cells.

### COX-2 inhibition attenuates *H. pylori*-induced effects on beta-catenin expression in SGC7901 and MKN45 cells

**Fig. 6:**
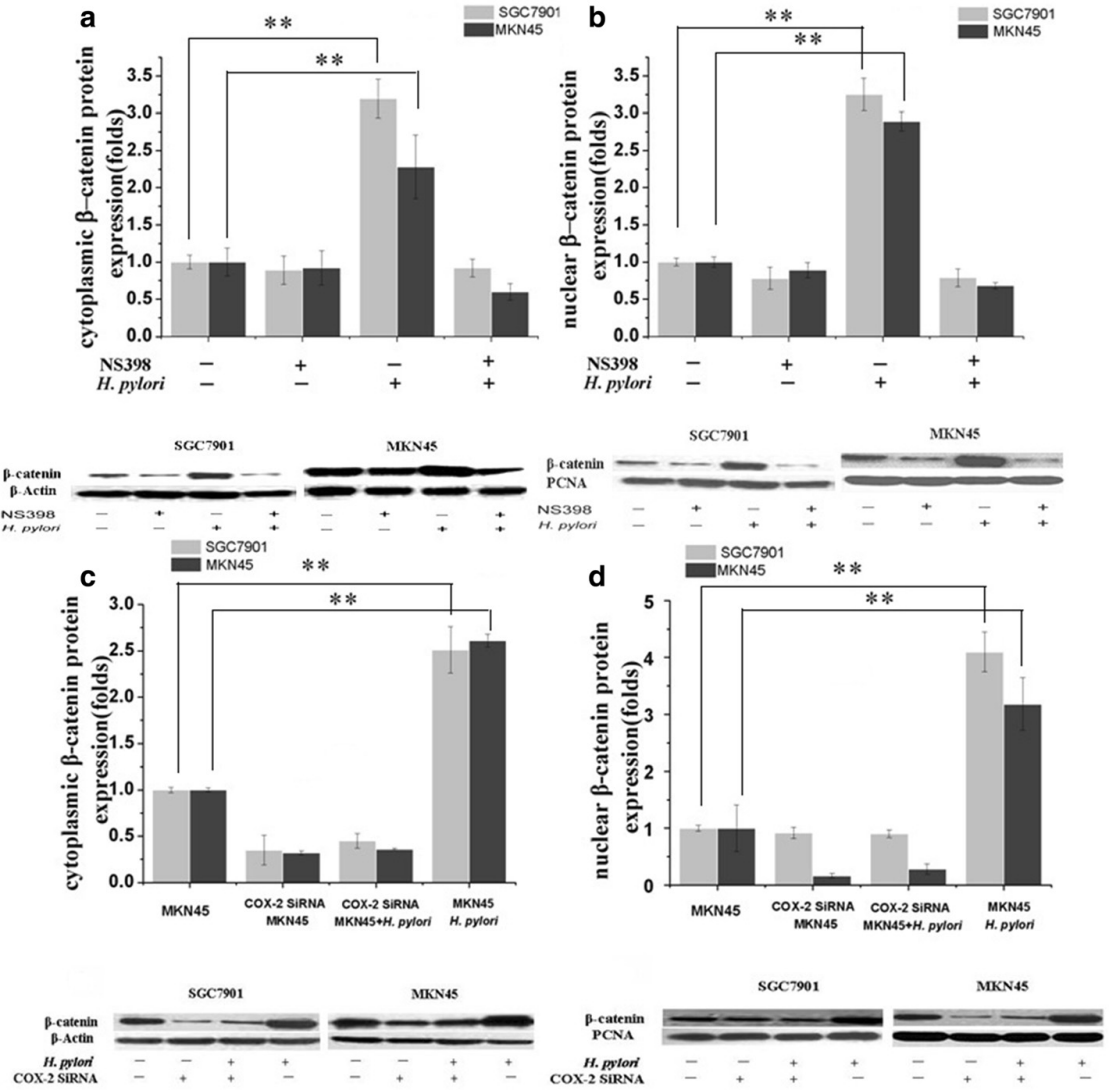
COX-2 inhibition attenuates *H. pylori*-induced beta-catenin upregulation in SGC7901 and MKN45 cells. **a**, **b**. COX-2 inhibitor NS398 attenuated *H. pylori* effects on cytoplasmic and nuclear beta-catenin protein expression. Confluent SGC7901 and MKN45 cells were pretreated with 50 μM NS398 for 2 h prior to culture with or without *H. pylori* for 12 h. Cytoplasmic and nuclear beta-catenin protein levels were then determined by western blotting. **P < 0.01 for *H. pylori*-infected versus control cells. **c**, **d** *COX2* RNAi blocked the *H. pylori* induced upregulation of cytoplasmic and nuclear beta-catenin. Confluent SGC7901 and MKN45 cells were infected with pFU-GW-COX-2-shRNA for 72 h with or without *H. pylori* incubation for 12 h. Cytoplasmic and nuclear beta-catenin protein expression levels were then measured by western blotting. **P < 0.01 for MKN45^+^ *H. pylori* versus MKN45 control and *COX2* siRNA^+^MKN45^+^ *H. pylori* cells

To determine whether COX-2 is responsible for Wnt/beta-catenin activation, SGC7901 and MKN45 cells were transfected with pFU-GW-COX-2-shRNA or the COX-2 inhibitor NS398 (50 μM, 2 h). After COX-2 gene silencing or COX-2 pathway inhibition, cytoplasmic and nuclear beta-catenin protein levels were significantly inhibited, showing that COX-2 is partly responsible for Wnt/beta-catenin activation (Fig. 6).

## Discussion

Malignant tumor growth and metastasis are complex processes related not only to the characteristics of the tumor itself but also to those of the tumor growth environment. Numerous studies have shown that increased MVD is an important feature of the tumor growth environment, which is a key factor in promoting tumor growth. *H. pylori*-induced angiogenesis in the gastric mucosa is important for the occurrence and development of gastric cancer. We first observed *H. pylori* colonization, COX-2, VEGF, beta-catenin levels and MVD in gastric cancer tissues. We found that approximately one third of gastric cancer tissues have *H. pylori* colonization, which is not a very high proportion. This suggests that many factors lead to gastric cancer development and *H. pylori* is only one of these. This may also explain why the gastric mucosa can no longer support *H. pylori* survival after gastric carcinogenesis. We also found that COX-2, VEGF, and beta-catenin expression and MVD were significantly higher in *H. pylori*-positive gastric cancer tissues than in *H. pylori*-negative gastric cancer tissues, indicating that *H. pylori* plays an important role in blood vessel formation in gastric cancer.

Some investigators have suggested an association between *H. pylori* infection and angiogenesis. Sasaki *et al*. previously reported tumor vascularity was greater in *H. pylori*-infected gastric cancer patients than in gastric cancer patients after *H. pylori* eradication [26]. *H. pylori* heat shock protein 60 (HSP60) enhances angiogenesis via CXCR2/PLCbeta2/Ca^2+^ signal transduction in HUVECs [27], but until now, the mechanism of *H. pylori* induced angiogenesis has remained poorly understood.

Tumor angiogenesis results from an imbalance between positive and negative angiogenic factors released by tumor and host cells into the neoplastic tissue microenvironment [28]. Gastric cancer cells produce many angiogenic factors, including VEGF, interleukin-8 and platelet-derived endothelial growth factor. Furthermore, *H. pylori* infection increases the expression of these angiogenic factors. Of these, VEGF is the key regulator of tumor-associated angiogenesis. The mechanism of *H. pylori-*induced VEGF upregulation is complicated and unclear. Our previous study found that the p38 MAPK-COX-2-EP2/EP4 axis regulates *H. pylori*-induced VEGF upregulation in gastric cells, providing a theoretical basis for investigating the pathogenesis of *H. pylori*-induced gastric cancer [25]. *H. pylori* also stimulates host VEGF gene expression via MEK/ERK-dependent activation of Sp1 and Sp3 [29]. Our findings suggest that infection with *H. pylori* standard strain NCTC11637 leads to a remarkable increase in VEGF expression in SGC7901 and MKN45 gastric epithelial cells via increasing the expression of COX-2, an important factor in gastric cancer development.

Many studies have shown that multiple intracellular pathways are activated by *H. pylori* [30–33], including the Wnt/beta-catenin pathway [34]. In the absence of Wnt signaling, beta-catenin is present in the cellular beta-catenin destruction complex. This multiprotein complex contains axin and adenomatous APC scaffolds that bind beta-catenin to facilitate its phosphorylation by casein kinase 1 (CKI) at Ser-45 and by GSK3beta at Ser-33, Ser-37, and Thr-41 [35]; it is targeted for degradation by the proteasome. Wnt pathway activation leads to depolymerization of the destruction complex, resulting in cytoplasmic beta-catenin accumulation and further transcription in the nucleus [36, 37]. The Wnt/beta-catenin pathway is involved in the development of a variety of malignant tumors, including gastric cancer [35, 38]. The *H pylori* cag secretion system activates beta-catenin, p120, and PPARδ, which promote gastric epithelial cell proliferation and might therefore contribute to gastric adenocarcinoma development in humans [39]. Studies have also shown that the Wnt/beta-catenin signaling plays an important role in gastric cancer angiogenesis. Beta-catenin is an important part of the angiogenesis pathway [40, 41]. However, the role of Wnt/beta-catenin in gastric cancer angiogenesis and the mechanism responsible for this remain to be elucidated.

Our results have demonstrated that *H. pylori* infection upregulates COX-2, which reduces cytoplasmic beta-catenin protein phosphorylation and induces cytoplasmic and nuclear beta-catenin accumulation, thus showing that *H. pylori* infection can induce the nuclear translocation of beta-catenin. We also confirmed that *H pylori* stimulates gastric epithelial cells to secrete VEGF, a proangiogenic factor, via the COX-2/Wnt/beta-catenin pathway, which may be an important drug target for preventing and treating *H. pylori* infection. Future studies should aim to determine which bacterial components induce angiogenesis in *H. pylori*-infected cells. Moreover, COX-2 and Wnt inhibitors have been used to treat tumors in clinical trials, and further studies may reveal whether these inhibitors can be used to treat gastric cancer [42].

## Conclusions

This study indicates *H. pylori* infection correlated with the depth of tumor invasion, degree of lymph node metastasis, and TNM stage. Moreover, *H. pylori* infection promoted COX-2, VEGF, and beta-catenin expression and increased MVD in gastric cancer. Further studies revealed that *H. pylori* infection induces VEGF upregulation via the COX-2/Wnt/beta-catenin pathway.

## Ethics approval and consent to participate

Human tissue is involved in the study, and the study was approved by the IRB of Shuguang Hospital, Shanghai University of TCM.

## Consent for publication

Not applicable.

## Availability of data and materials

We state that data will not be shared. The National Natural Science Foundation of China (81273958) has not yet been completed, so the date is not open.

## Abbreviations

ANOVA: analysis of variance
APC: adenomatous polyposis coli
ATF-2: activating transcription factor-2
ATF-2: adenomatous polyposis coli
BCA: bicinchoninic acid
CA: State of California
COX-2: cyclooxygenase 2
DMEM: Dulbecco’s modified eagle medium
ELISA: enzyme linked immunosorbent assay
FAM: 6-carboxy-fluo-rescein-phosphoramidite
GSK3beta: glycogen synthase kinase 3 beta
*H. pylori*: *Helicobacter pylori*
HSP60: heat shock protein 60
IARC: International Agency for Research on Cancer
LEF1/TCF: lymphoid-enhancing factor/T-cell factors
MAPK: mitogen-activated protein kinases
MVD: microvessel density
NIH: National Institutes of Health
OD: optical density
PBS: phosphate-buffered saline
PE Biosystems: PerkinElmer Biosystems
PVDF: polyvinylidene fluoride
RFQ-PCR: real-time fluorogenic quantitative polymerase chain reaction
RNA: ribonucleic acid
RPMI: Roswell Park Memorial Institute
RT-PCR: reverse transcription polymerase chain reaction
SE: standard error
TAMPA: carboxy-tetramethyl-rhodamine
TBM: 3,3′,5,5′-Tetramethylbenzidine
TBS: tris-buffered saline
USA: United States of America
VEGF: vascular endothelial growth factor
WHO: World Health Organization
